# Supplementary material for: Integrated analyses of growth differentiation factor-15 concentration and cardiometabolic diseases in humans
Source: eLife. 2022 Aug 2;11:e76272. doi: 10.7554/eLife.76272 (PMC9391041; doi:10.7554/eLife.76272)
Supplement: Supplementary file 1. — (a) FINRISK cohort characteristics. (b) Baseline characteristics in FINRISK associated with growth differentiation factor-15 (GDF15) plasma levels. [file elife-76272-supp1.docx]

**Supplementary file 1a. FINRISK cohort characteristics.**

|  | **Overall**  **(N= 6610)** | **Female**  **(N=3257)** | **Male**  **(N= 3353)** |
| --- | --- | --- | --- |
| *GDF15*  *(ng/L, mean, ± SD, range)* | 1053 (2046) | 1166 (2858) | 943 (542) |
| *Baseline age*  *(years, mean, ± SD, range)* | 47.7 (13.1) | 46.2 (12.6) | 49.2 (13.4) |
| **Cardiometabolic risk factors** |  |  |  |
| *Smoking (%, n)* | 26.1 (6555) | 21.0 (3237) | 31.1 (3318) |
| *Body mass Index*  *(kg/m^2^; mean, ± SD, range, n)* | 26.6 (4.5, 6602) | 25.3 (5.0, 3254) | 27.0 (4.0, 3348) |
| *Systolic Blood Pressure*  *(mmHg; mean, ± SD, range, n)* | 136 (19, 6604) | 131 (19, 3254) | 140 (19, 3350) |
| *Total Cholesterol*  *(mmol/L; mean, ± SD, range)* | 5.5 (1.1) | 5.5 (1.1) | 5.6 (1.0) |
| *Triglycerides*  *(mmol/L; mean, ± SD, range)* | 1.5 (1.1) | 1.3 (0.9) | 1.7 (1.1) |
|  | **Overall**  **(N=6600)** | **Female**  **(N=3248)** | **Male**  **(N= 3352)** |
| **Outcome**  *All-cause mortality (%)* | 16.4 | 10.3 | 22.3 |
| *Type 2 Diabetes (%)* | 8.9 | 6.7 | 10.5 |
| *Myocardial infarction (%)* | 7.2 | 3.7 | 10.5 |
| *Heart Failure (%)* | 6.2 | 4.4 | 8.0 |
| *Cardiovascular disease (%)* | 51.7 | 50.1 | 52.1 |

**Supplementary file 1b. Baseline characteristics in FINRISK associated with GDF15 plasma levels.**

|  | **Beta (SE)** | ***p*-value** | **Variance of GDF15 explained (%)** |
| --- | --- | --- | --- |
| **Age** | 0.041 (0.00080) | < 2.2x10^-308^ | 28.0 |
| **Gender** | -0.033 (se=0.021) | 0.12 | - |
| **BMI** | 0.039 (se=0.011) | 0.00051 | 0.1 |
| **Smoking** | 0.34 (0.024) | 2.2x10^-44^ | 1.9 |
