# Supplementary material for: Integrated analyses of growth differentiation factor-15 concentration and cardiometabolic diseases in humans
Source: eLife. 2022 Aug 2;11:e76272. doi: 10.7554/eLife.76272 (PMC9391041; doi:10.7554/eLife.76272)
Supplement: Transparent reporting form [file elife-76272-transrepform1.docx]

***eLife’s* transparent reporting form**

We encourage authors to provide detailed information *within their submission* to facilitate the interpretation and replication of experiments. Authors can upload supporting documentation to indicate the use of appropriate reporting guidelines for health-related research (see [EQUATOR Network](http://www.equator-network.org/ )), life science research (see the [BioSharing Information Resource](https://biosharing.org/)), or the [ARRIVE guidelines](http://www.plosbiology.org/article/info:doi/10.1371/journal.pbio.1000412) for reporting work involving animal research. Where applicable, authors should refer to any relevant reporting standards documents in this form.

If you have any questions, please consult our Journal Policies and/or contact us: [editorial@elifesciences.org](mailto:editorial@elifesciences.org).

**Sample-size estimation**

- You should state whether an appropriate sample size was computed when the study was being designed
- You should state the statistical method of sample size computation and any required assumptions
- If no explicit power analysis was used, you should describe how you decided what sample (replicate) size (number) to use

Please outline where this information can be found within the submission (e.g., sections or figure legends), or explain why this information doesn’t apply to your submission:

All information on the methods used are detailed in the Materials and Methods section (page 24).

- Sample sizes are provided in our manuscript at lines 453, 467, 471, 555 and 687.
- No power analyses were conducted. Instead utilised all available data and meta-analysed the results from 3 cohorts to maximise power.

**Replicates**

- You should report how often each experiment was performed
- You should include a definition of biological versus technical replication
- The data obtained should be provided and sufficient information should be provided to indicate the number of independent biological and/or technical replicates
- If you encountered any outliers, you should describe how these were handled
- Criteria for exclusion/inclusion of data should be clearly stated
- High-throughput sequence data should be uploaded before submission, with a private link for reviewers provided (these are available from both GEO and ArrayExpress)

Please outline where this information can be found within the submission (e.g., sections or figure legends), or explain why this information doesn’t apply to your submission:

All information can be found in the Materials and Methods (page 24), Results (page 6).

- Data from the genome-wide association studies of GDF15 levels were replicated across different assay platforms and independent cohorts and then meta-analysed (Results subsection: “Genome-wide association studies of plasma levels of GDF15 quantified using 3 different assays in 2 independent cohorts”, Methods subsection: “Meta-analysis”).
- We did not remove any outliers in the biomarker analysis to ensure no data that could be of biological relevance was removed (Results subsection “Association of GDF15 plasma levels with 676 disease outcomes, Methods subsection “Study population and phenotypes; FINRISK”).

**Statistical reporting**

- Statistical analysis methods should be described and justified
- Raw data should be presented in figures whenever informative to do so (typically when N per group is less than 10)
- For each experiment, you should identify the statistical tests used, exact values of N, definitions of center, methods of multiple test correction, and dispersion and precision measures (e.g., mean, median, SD, SEM, confidence intervals; and, for the major substantive results, a measure of effect size (e.g., Pearson's r, Cohen's d)
- Report exact p-values wherever possible alongside the summary statistics and 95% confidence intervals. These should be reported for all key questions and not only when the p-value is less than 0.05.

Please outline where this information can be found within the submission (e.g., sections or figure legends), or explain why this information doesn’t apply to your submission:

All information can be found in the Materials and Methods (page 24), Results (page 6) and Supplementary Materials (separate files).

- Each statistical method is outlined in the Materials and Methods section with descriptions of N values, multiple-testing correction and precision measures.
- Raw data is shown in Figures throughout the main text.
- All raw p-values for results are provided in the main text in the Results section (Tables 2, 3 and 4) and in the Supplementary Tables when not present in the main text.

(For large datasets, or papers with a very large number of statistical tests, you may upload a single table file with tests, Ns, etc., with reference to sections in the manuscript.)

**Group allocation**

- Indicate how samples were allocated into experimental groups (in the case of clinical studies, please specify allocation to treatment method); if randomization was used, please also state if restricted randomization was applied
- Indicate if masking was used during group allocation, data collection and/or data analysis

Please outline where this information can be found within the submission (e.g., sections or figure legends), or explain why this information doesn’t apply to your submission:

All information can be found in the Materials and Methods (page 24), Results (page 6).

- Randomisation was not used as groups were defined by clinical doctor notes and by original study (Methods subsection “Study population and phenotypes”).
- Participants in the studies were given a random study ID and were not identifiable (consent is listed in Methods subsection “Study population and phenotypes”).

**Additional data files (“source data”)**

- We encourage you to upload relevant additional data files, such as numerical data that are represented as a graph in a figure, or as a summary table
- Where provided, these should be in the most useful format, and they can be uploaded as “Source data” files linked to a main figure or table
- Include model definition files including the full list of parameters used
- Include code used for data analysis (e.g., R, MatLab)
- Avoid stating that data files are “available upon request”

Please indicate the figures or tables for which source data files have been provided:

- Tables 1-4 in the main text show summary data from the analysis. Further summary results are shown in full in the Supplementary Tables.
- Only well-established statitistical methods were applied in R. All R versions and packages are stated in the Materials and Methods section (line 529, 531, 558, 611, 669, 670 and 703)
